# Cannabis use and involuntary admission may mediate long-term adherence in first-episode psychosis patients: a prospective longitudinal study

**DOI:** 10.1186/1471-244X-13-326

**Published:** 2013-12-01

**Authors:** Sara Barbeito, Patricia Vega, Sonia Ruiz de Azúa, Margarita Saenz, Mónica Martinez-Cengotitabengoa, Itxaso González-Ortega, Cristina Bermudez, Margarita Hernanz, Blanca Fernández de Corres, Ana González-Pinto

**Affiliations:** 1 Biomedical Research Centre in Mental Health Net (CIBERSAM), University Hospital of Álava, Olaguibel Street, Vitoria, Spain; 2 National University of Distance Education (Universidad Nacional de Educación a Distancia; UNED), Madrid, Spain; 3 Basque Country University, Sarrienas/n, 48940 Leioa, Bizkaia, Spain; 4 Department of Psychiatry, CIBERSAM G10, Hospital Santiago, Olaguibel 29, Vitoria, Spain

**Keywords:** Adherence, Cannabis, First psychotic episode, Involuntary admission

## Abstract

**Background:**

This study aimed to examine factors associated with treatment adherence in first-episode psychosis (FEP) patients followed up over 8 years, especially involuntary first admission and stopping cannabis use.

**Methods:**

This prospective, longitudinal study of FEP patients collected data on symptoms, adherence, functioning, and substance use. Adherence to treatment was the main outcome variable and was categorized as ‘good’ or ‘bad’. Cannabis use during follow-up was stratified as continued use, stopped use, and never used. Bivariate and logistic regression models identified factors significantly associated with adherence and changes in adherence over the 8-year follow-up period.

**Results:**

Of the 98 FEP patients analyzed at baseline, 57.1% had involuntary first admission, 74.4% bad adherence, and 52% cannabis use. Good adherence at baseline was associated with Global Assessment of Functioning score (*p* = 0.019), Hamilton Depression Rating Scale score (*p* = 0.017) and voluntary admission (*p* < 0.001). Adherence patterns over 8 years included: 43.4% patients always bad, 26.1% always good, 25% improved from bad to good. Among the improved adherence group, 95.7% had involuntary first admission and 38.9% stopped cannabis use. In the subgroup of patients with bad adherence at baseline, involuntary first admission and quitting cannabis use during follow up were associated with improved adherence.

**Conclusions:**

The long-term association between treatment adherence and type of first admission and cannabis use in FEP patients suggest targets for intervention to improve clinical outcomes.

## Background

First-episode psychosis (FEP) patients often demonstrate denial of illness, refusal to be admitted into a psychiatric unit, and poor medication adherence. As many as 60% of FEP patients have poor adherence to medication [1], and 20% persistently refuse medication [2]. FEP patients often require inpatient care and the type of hospital admission (voluntary or involuntary) may be associated with treatment adherence and with the severity and/or prognosis of the disorder [3].

Factors influencing adherence in FEP patients have been examined in only a few prospective longitudinal studies and the findings have been inconsistent. Among factors reported to have a negative effect on adherence are greater symptom severity (positive symptoms, especially delusions and paranoia), hostility, poorer cognitive functioning, lower parental social class, less education, medication side-effects, longer duration of untreated psychosis, substance abuse, alcohol consumption, and poor insight [1,4-7]. On the other hand, a good patient‒doctor/therapist alliance has been associated with positive effects on adherence [8].

Patient characteristics associated with involuntary first admission include worse psychopathology (positive symptoms), suicidal behavior, aggressive behavior, lack of insight, substance use, and worse functioning [3,9-11]. However, outcome differences between patients admitted voluntarily and involuntarily and the factors associated with these outcomes have not been well studied. There is some evidence that patients admitted involuntarily have poorer long-term outcomes because they are less likely to use medication and to engage with mental health services after hospital discharge than patients admitted voluntarily [12]. In one study of FEP patients, involuntary admission was an independent predictor of medication adherence over 5 years of follow-up [4]. Conversely, in another study, treatment adherence, psychopathology and functioning at the 2-year follow-up did not differ between FEP patients admitted voluntarily and involuntarily [3]. Nevertheless, most patients admitted involuntarily eventually agree that forced admission was necessary at the time and that their treatment was beneficial, and these perceptions are associated with better insight [13,14].

Substance use, especially cannabis use, is common among patients with psychosis [15], and is associated with worse clinical outcomes, including increased relapse rates [16]. Reported rates of substance abuse in FEP patients vary (ranging from 11% to 62%), and patients with persistent substance use have an increased risk of relapse/readmission and poorer functional outcome than those who stop using substances or who have never used them [17]. Cannabis use may be a risk factor for non-adherence or discontinuation of treatment in FEP patients [18], and was consistently associated with non-adherence in a systematic review [16]. The long-term consequences of poor adherence in FEP patients include more frequent episodes and hospital readmissions, especially involuntary admissions [13], and a higher risk of suicide [19]. Early identification of factors that predict poor adherence and, consequently, a worse course of illness would facilitate the early use of beneficial interventions. Enhanced adherence in the first few years of illness may improve medium- and long-term outcomes [20]. Long-term studies in FEP patients are needed.

The main aim of this prospective longitudinal study was to identify factors associated with adherence to treatment in FEP patients, with a particular focus on whether the type of first hospitalization (voluntary versus involuntary), other baseline factors, and cannabis use over the 8 year follow-up period were important variables affecting adherence. We also examined factors associated with the change in adherence over 8 years of follow-up, and specifically assessed whether the change in adherence for patients with bad adherence at baseline was associated with type of first hospitalization (voluntary versus involuntary) and change in cannabis use over 8 years of follow-up. We hypothesized that (a) adherence in the long-term is influenced by both the type of first hospitalization and cannabis use during follow-up, and (b) FEP patients with involuntary first admission and/or who stop using cannabis may have improved adherence in the long-term.

## Methods

### Patients and methods

Data were gathered on recent-onset FEP patients admitted consecutively to a general hospital psychiatric ward between 1997 and 1999, and who were all subsequently followed-up over 8 years. The hospital provides psychiatric care to all inhabitants (approximately 300 000 people) of the catchment area of the city of Vitoria in the Spanish Basque Country. As there are no other psychiatric inpatient units in this area, the study sample is representative of FEP patients who need inpatient psychiatric care. The study was approved by the Ethics Committee of Hospital Santiago Apóstol and the participating patients were enrolled after providing informed consent.

The FEP patients included in the study were aged 18–65 years and met the DSM-IV criteria for schizophreniform disorder, schizoaffective disorder, schizophrenia, delusional disorder, brief psychotic disorder, psychotic disorder not otherwise specified (NOS), bipolar disorder with psychotic features, or major depressive disorder with psychotic features. Patients with substance-induced psychotic disorders, mental retardation or organic brain disorders were excluded from this study. The DSM-IV axis I diagnosis was made using the Structured Clinical Interview for DSM-IV (SCID-I) [21]. A first psychotic episode was defined as the first time a patient displayed positive psychotic symptoms of delusions and/or hallucinations or marked disorganized behavior. Admission status at first psychiatric hospitalization was defined as involuntary if the patient refused to be admitted to the unit and a judicial report was necessary.

### Assessments

Upon first hospital admission (baseline), patients with FEP symptoms were assessed using a protocol that included the SCID-I, urine drug screens (including cannabis derivatives), the Addiction Severity Index (ASI) (see Table 1), Positive and Negative Symptoms Scale (PANSS) [22,23], Hamilton Depression Rating Scale (HDRS-21) [24,25], Young Mania Rating Scale (YMRS) [26], Phillips Premorbid Adjustment scale [27], Global Assessment of Functioning (GAF) [28], and Strauss-Carpenter prognostic scale [29]. The same protocol was administered to all patients at 1, 2, 3, 4, 5 and 8 years follow-up. Some patients had further hospitalizations whereas others did not. During the 8-year follow-up period, all patients enrolled in the study were contacted and interviewed, and we reviewed their psychiatric and medical history.

**Table 1:** Information used to define the four categories of cannabis use

**Cannabis use category**	**DSM-IV abuse or dependence**	**Addiction Severity Index (ASI) score**
Dependence	Meet minimal or more DSM-IV criteria for cannabis dependence	8–9: extreme problem, treatment absolutely necessary
Abuse	Meet ≥1 criteria for cannabis abuse	4–5: moderate problem, some treatment indicated
		6–7: considerable problem, treatment necessary
Use	Meet abuse criteria but do not meet temporal criteria (at least 12 months) or use for 12 months but not fulfilling any criteria of DSM-IV abuse	2–3: slight problem, substance abuse treatment probably not necessary
No use	No significant symptoms	0–1: no real problem

Note: DSM-IV, Diagnostic Statistical Manual of Mental Disorders (fourth edition).

For each patient, relevant clinical and demographic variables were collected, and use and abuse of cannabis and other drugs was determined using information obtained from the patient, key informant, medical record, and drug screens. We determined whether cannabis or other substances had been used, how often they had been used, and when that use had occurred. Cannabis use was defined as having taken cannabis at least once in the month prior to admission, and at least 4 times in the last year. Cannabis use, abuse and dependence were defined according to DSM-IV, using SCID-I [21], and the data derived from urine analyses and the ASI (see Table 1). The same method was used to establish use, abuse or dependence of other drugs and alcohol. The research team members who determined drug use were blind to the clinical ratings; they discussed any inconsistencies and selected the most reliable source. At every year of follow-up, patients were also interviewed using the SCID-I and ASI, and urine analyses were performed.

Evaluations were performed during clinical interviews and pertained to the previous week. Interviews were conducted by three psychiatrists (A. G-P., S.B. and P.V.) who had good inter-rater reliability for SCID-I diagnoses (kappa = 0.88), and for the scales used (PANSS, kappa = 0.80; GAF, kappa = 0.95; Phillips premorbid scale, kappa = 0.77; YMRS, kappa = 0.83; HDRS-21, kappa 0.79; and Strauss–Carpenter, kappa = 0. 79).

Adherence was defined as the medication behavior of the patient (i.e., whether or not they took their medication regularly following medical advice). Patient adherence to treatment at baseline and during follow-up was evaluated using the Morisky–Green test, a validated, self-report, 4-item scale [30]. Patients answer yes (score = 0) or no (score = 1) to the following questions: “Do you sometimes forget to take your medicine?”, “Are you sometimes careless with the time you take your medicine?”, “When you feel good, do you sometimes not take your medicine?”, and “When you feel bad with the medicine, do you sometimes not take it?”. Patients with a score of 4 were considered as having ‘good’ adherence, while those with a score between 0 and 3 were classified as having ‘bad’ adherence. Previous studies have demonstrated the validity and usefulness of this self-report tool [30,31].

At each visit, the number of relapses since the previous visit was recorded together with scores for GAF-evaluated psychosocial functioning, Clinical Global Impression (CGI), and the Strauss–Carpenter prognostic scale. The Strauss–Carpenter subscales collected information on hospitalizations in the last 12 months, work, social activity, global functioning and symptoms in the last month, with a lower score indicating a worse patient status. The number of hospitalizations and suicide attempts over the 8-year follow-up was recorded. Additional information from clinical records, psychiatrists, family informants, and staff observations was obtained as necessary. Patients who could not be contacted during the study period were considered lost to follow-up.

All patients received pharmacological treatment, mainly atypical antipsychotics, with no bias regarding their cannabis use or not. After hospital discharge, patients received standard care at their community mental health center, which was usually one visit per month. Family interventions and psychological support were provided if required. Further care was prescribed as necessary, including hospitalizations, which were independent of patient socioeconomic status. If immediate attention was needed, an emergency room was available on a 24-hour basis.

### Patient classification

Patients were classified as having voluntary or involuntary first admission to the psychiatric hospital (baseline). Adherence to treatment at each visit was categorized as ‘good’ or ‘bad’ based on the Morisky–Green test scores, and we present the data for adherence (good/bad) at the baseline visit and at the 8-year visit. In addition, the change in adherence over 8 years of follow-up was categorized as ‘always bad’, ‘always good’, ‘improved from bad to good’, or ‘worsened from good to bad’. For this, we considered the adherence rating (good/bad) at baseline and at each visit from year 1 to year 8. Each patient’s pattern of cannabis use over the 8-year follow-up period was classified as: ‘stopped use’, ‘continued use’ (i.e., the patient used cannabis before the baseline evaluation and continued to use it throughout the follow-up period) or ‘never used’.

### Statistical analyses

Comparisons between the good and bad adherence groups were made using bivariate analyses: Mann–Whitney *U* tests for continuous variables and Chi-square tests for categorical variables. A linear regression analysis was performed to assess the relationship between adherence at the 8-year visit (good or bad) and cannabis use during follow-up (stopped use or never use versus continued use).

A logistic regression model was used to analyze the association between adherence at 8 years (bad versus good) and basal clinical variables. Finally, a multiple logistic regression model determined factors associated with the change in adherence (always bad versus improved) during 8 years of follow-up; cannabis use (stopped use or never use versus continued use) during the study and type of hospitalization (involuntary versus voluntary) were both included as independent variables in the model.

Data are presented as beta coefficients and odds ratios (OR) with *p* values.

All data were analyzed using SPSS 21.0 version.

## Results

### Baseline characteristics

Of the 98 FEP patients who met the study inclusion criteria, were enrolled at baseline, and included in the analysis, six died during follow-up.

The baseline characteristics of the study sample (Table 2) show that admission status was voluntary for 42 (42.9%) patients and involuntary for 56 (57.1%) patients. All patients with involuntary admission had bad adherence at baseline (*p* < 0.001 versus good adherence).

**Table 2:** Baseline characteristics of the total sample of FEP patients and by baseline adherence (good, bad)

**Variable**	**Total sample (*N*** **= 98)**	**Bad adherence (*n*** **= 73)**	**Good adherence (*n*** **= 25)**	***p *** **value**
Gender, *n* (%)				Chi^2^ = 0.003 (*p* = 0.954)
Male	71 (72.4)	53 (72.6)	18 (72.0)	
Female	27 (27.6)	20 (27.4)	7 (28.0)	
Age	29.8 ± 10.7	29.9 ± 10.2	30.7 ± 12.2	*U* = 762.5 (*p* = 0.858)
Civil status, *n* (%)				Chi^2^ = 2.910 (*p* = 0.233)
Single	74 (75.5)	57 (78.1)	17 (68.0)	
Married	17 (17.3)	10 (13.7)	7 (28.0)	
Other	7 (7.2)	6 (8.2)	1 (4.0)	
Family history, *n* (%)				Chi^2^ = 6.036 (***p*** **= 0.014**)
No	16 (16.3)	8 (10.9)	8 (32.0)	
Yes	82 (83.7)	65 (89.1)	17 (68.0)	
Diagnosis, *n* (%)				Chi^2^ = 0.601 (*p* = 0.740)
Bipolar disorder & affective	33 (33.7)	23 (31.5)	10 (40.0)	
Schizophrenia	39 (39.8)	30 (41.1)	9 (36.0)	
Other	26 (26.5)	20 (27.4)	6 (24.0)	
PANSS basal positive^a^	24.8 ± 6.9	25.0 ± 6.7	24.4 ± 8.0	*U* = 636.0 (*p* = 0.640)
PANSS basal negative^a^	18.9 ± 9.4	18.3 ± 9.1	20.5 ± 9.7	*U* = 598.0 (*p* = 0.395)
PANSS basal general^a^	42.2 ± 11.4	40.6 ± 10.4	47.7 ± 13.1	*U* = 598.0 (*p* = 0.395)
GAF basal^b^	55.5 ± 17.9	58.5 ± 16.2	69.2 ± 14.8	*U* = 152.5 (***p*** **= 0.019**)
YMRS basal	25.4 ± 12.1	26.8 ± 12.4	21.6 ± 11.8	*U* = 494.5 (*p* = 0.059)
HDRS-21 basal	17.9 ± 8.4	16.4 ± 7.0	22.6 ± 10.9	*U* = 446.0 (***p*** **= 0.017**)
Phillips total basal^c^	5.6 ± 3.0	5.9 ± 2.9	5.2 ± 3.5	*U* = 587.5 (*p* = 0.337)
Strauss total basal^b^	12.8 ± 3.9	12.5 ± 3.7	13.1 ± 4.5	*U* = 281.5 (*p* = 0.830)
Aggressiveness, *n* % yes [vs. no]	73 (74.5)	57 (78.1)	16 (64.0)	Chi^2^ = 1.943 (*p* = 0.163)
Tobacco use, *n* (%) yes [vs. no]	79 (80.6)	61 (83.6)	18 (72.0)	Chi^2^ = 1.593 (*p* = 0.207)
Alcohol use, *n* (%) yes [vs. no]	94 (95.9)	72 (98.6)	22 (88.0)	Chi^2^ = 5.375 (***p*** **= 0.020**)
Cannabis use, *n* (%) yes [vs. no]	51 (52.0)	40 (54.8)	11 (44.0)	Chi^2^ = 0.869 (*p* = 0.351)
Other drugs use, *n* (%) yes [vs. no]	33 (33.7)	25 (34.2)	8 (32.0)	Chi^2^ = 0.042 (*p* = 0.837)
Voluntary admission, *n* (%)				Chi^2^ = 44.749 (***p*** **< 0.001**)
No	56 (57.1)	56 (76.7)	0 (0)	
Yes	42 (42.9)	17 (23.3)	25 (100)	

Data are presented as mean ± SD or *n* (%).

Total *n* varies for the different variables due to missing data. The percentage given for each variable refers to the total *n* available for that variable.

*P* values are results of Chi-square (categorical variables) and Mann–Whitney *U* tests (continuous variables) for the comparison between groups with good versus bad adherence.

Values in bold are significant at *p* < 0.05.

Note: PANSS, Positive and Negative Symptoms Scale; GAF, Global Assessment of Functioning; YMRS, Young Mania Rating Scale; HDRS-21, Hamilton Depression Rating Scale, Phillips total; Phillips Premorbid adjustment scale total score; Strauss, Strauss-Carpenter prognostic scale score.

^a^For PANSS scores, a higher score indicates worse symptoms.

^b^For GAF and Strauss, a lower score indicates poorer functioning.

^c^For Phillips, a higher score indicates poorer premorbid adjustment.

Adherence was bad for 74.4% (73/98) of patients and good for 25.5% (25/98) of patients at baseline, with few significant differences in characteristics between the good and bad adherence groups, except for family history of psychosis (Table 2). Patients with good adherence at baseline had higher mean scores for GAF (*p* = 0.019) and HDRS-21 (*p* = 0.017), and were more likely to have voluntary admission (*p* < 0.001) than the bad adherence group. The Strauss–Carpenter total score did not differ between adherence groups at baseline.

The total sample had a high frequency of alcohol consumption (95.9%), tobacco use (80.6%) and cannabis use (52%). There was no significant difference between adherence groups in these variables, except for a higher consumption of alcohol in the bad adherence subgroup (Table 2).

### 8-Year data

#### - Adherence (basal and at 8 years) and clinical and functional variables at 8 years

The percentage of patients with good adherence increased from 25.5% (25/98) at baseline to 51.1% (47/92) at the 8-year visit. In the logistic regression model, adherence at 8 years was not associated with any of the clinical variables (PANSS, GAF, YMRS, HDRS, Phillips, Strauss‒Carpenter) except for cannabis use during follow-up. Type of first hospitalization (voluntary/involuntary) was not associated with adherence at the 8-year visit (B = 0.456; OR = 1.578; *p* = 0.314).

The bivariate analysis in Table 3 shows that patients with good adherence at 8 years had better outcomes than patients with bad adherence at 8 years, with a higher mean GAF score at 8 years (*p* = 0.007), a lower YMRS score at 8 years (*p* = 0.013), a higher Strauss–Carpenter total score at 8 years (*p* = 0.010), less hospitalization in the previous year (*p* = 0.001), better working capacity (*p* = 0.007), fewer symptoms in the previous month (*p* < 0.001), and better global functioning in the previous year (*p* < 0.001). There were no significant differences between patients with good and bad adherence at 8 years for the PANSS scores (Table 3).

**Table 3:** Clinical characteristics of total study sample at 8-year visit and by adherence at 8-year visit

**Variable**	**Total sample (*N*** **= 92)**	**Bad adherence (*n*** **= 45)**	**Good adherence (*n*** **= 47)**	***p *** **value**
PANSS positive	24.8 ± 6.9	25.2 ± 7.8	24.4 ± 6.4	U = 607.0 (*p* = 0.413)
PANSS negative	18.9 ± 9.4	18.9 ± 9.4	20.0 ± 9.8	U = 652.5 (*p* = 0.745)
PANSS general	42.2 ± 11.4	44.0 ± 10.7	42.5 ± 11.9	U = 617.0 (*p* = 0.478)
YMRS 8 years	8.0 ± 9.2	12.0 ± 10.8	5.5 ± 7.2	U = 304.00 **(*p*** **= 0.013)**
Phillips 8 years	6.3 ± 2.9	6.9 ± 2.5	5.6 ± 3.0	U = 354.50 (*p* = 0.510)
GAF 8 years	61.3 ± 16.8	56.2 ±16.1	68.1 ± 15.9	U = 192.00 (***p*** **= 0.007)**
Strauss 8 year total	12.8 ± 3.4	12.2 ± 4.6	15.2 ± 4.7	U = 189.5 (***p*** **= 0.010**)
Strauss hospitalization, *n* (%) yes [vs. no]	35 (37.7)	25 (53.8)	10 (19.2)	Chi^2^ = 11.461 (***p*** **= 0.001**)
Strauss work, *n* (%)				Chi^2^ = 9.809 (***p*** **= 0.007**)
No	41 (44.6)	24 (53.3)	17 (36.2)	
Partial	14 (15.2)	10 (22.2)	4 (8.5)	
Total	37 (40.2)	11 (24.5)	26 (55.3)	
Strauss social activity, *n* (%)				Chi^2^ = 1.595 (*p* = 0.450)
No	14 (15.2)	7 (15.6)	7 (14.9)	
Little contact	7 (7.6)	5 (11.1)	2 (4.2)	
Frequently	71 (77.2)	33 (73.3)	38 (80.9)	
Strauss symptoms, *n* (%)				Chi^2^ = 25.641 (***p*** **< 0.001**)
Severe	14 (15.2)	8 (17.8)	6 (12.8)	
Moderate	20 (21.7)	18 (40.0)	2 (4.2)	
Light	32 (34.8)	15 (33.3)	17 (36.2)	
None	26 (28.3)	4 (8.9)	22 (46.8)	
Strauss global functioning, *n* (%)				Chi^2^ = 22.561 (***p*** **< 0.001**)
Continued & severe	2 (2.2)	2 (4.4)	0 (0)	
Important	11 (12.0)	6 (13.3)	5 (10.6)	
Moderate	27 (29.3)	21 (46.7)	6 (12.8)	
Light	30 (32.6)	13 (28.9)	17 (36.2)	
No disorder	22 (23.9)	3 (6.7)	19 (40.4)	
Alcohol use, *n* (%) yes [vs. no]	52 (56.5)	31 (68.9)	21 (44.7)	Chi^2^ = 5.482 (***p*** **= 0.019**)
Cannabis use, *n* (%) yes [vs. no]	27 (29.3)	21 (46.7)	6 (12.8)	Chi^2^ = 12.742 (***p*** **< 0.001**)
Other drugs use, *n* (%) yes [vs. no]	15 (16.3)	15 (33.3)	0 (0)	Chi^2^ = 18.719 (***p*** **< 0.001**)
Evolution of cannabis use, *n* (%)				Chi^2^ = 11.436 (***p*** **= 0.003**)
Stop use	25 (27.2)	9 (20.0)	16 (34.0)	
Continue use	26 (28.3)	20 (44.4)	6 (12.8)	
Never use	41 (44.5)	16 (35.6)	25 (53.2)	

Data are presented as mean ± SD or *n* (%).

Total *n* varies for the different variables due to missing data. The percentage given for each variable refers to the total *n* available for that variable.

*p* values are results of Chi-square (categorical variables) and Mann–Whitney *U* tests (continuous variables) for the comparison between groups with good versus bad adherence.

Values in bold are significant at *p* < 0.05.

Note: PANSS, Positive and Negative Symptoms Scale; GAF, Global Assessment of Functioning; YMRS, Young Mania Rating Scale; HDRS-21, Hamilton Depression Rating Scale, Phillips total; Phillips Premorbid adjustment scale total score; Strauss, Strauss-Carpenter prognostic scale score.

#### - Associations between adherence at 8 years and substance use

At 8 years (Table 3), the frequency of alcohol consumption (56.5%), cannabis use (29.3%) and use of other drugs (16.3%) had decreased from baseline (see Table 2). Compared with the bad adherence group, a significantly lower proportion of patients with good adherence at 8 years had alcohol consumption (*p* = 0.019), cannabis use (*p* < 0.001), or use of other drugs (*p* < 0.001).

Of the patients with bad adherence at 8 years, 44.4% (20/45) continued to use cannabis at 8 years, while only 12.8% (6/47) of patients with good adherence continued using cannabis (Table 3).

#### - Change in adherence over 8 years: associations with type of hospitalization and cannabis use

Table 4 shows that 70.0% (28/40) of patients with ‘always bad’ adherence had involuntary admission at baseline, as did 95.7% (22/23) of the patients with adherence that ‘improved from bad to good’ during the study.

**Table 4:** Patient variables associated with change in adherence over 8 years of follow-up

**Variable**	**Total sample (*N*** **= 92)**	**Adherence pattern over 8 years of follow-up**				**Chi**^**2 **^**value (*p *** **value)**
		**Always bad (*n*** **= 40)**	**Always good (*n*** **= 24)**	**Improved from bad to good (*n*** **= 23)**	**Worsened from good to bad (*n*** **= 5)**	
Voluntary admission at baseline, *n* (%)^a^						54.289 (***p*** **< 0.001**)
No	50 (54.3)	28 (70.0)	0 (0.0)	22 (95.7)	0 (0.0)	
Yes	42 (45.7)	12 (30.0)	24 (100.0)	1 (4.3)	5 (100.0)	
Evolution of cannabis use, *n* (%)						24.036 (***p*** **= 0.001**)
Stop use	23 (25.3)	4 (11.8)	6 (27.8)	9 (38.9)	4 (75.0)	
Continue use	27 (29.1)	20 (50.0)	4 (11.1)	3 (11.1)	0 (0.0)	
Never use	42 (45.6)	16 (38.2)	14 (61.1)	11 (50.0)	1 (25.0)	
GAF at 8 years	55.5 ± 17.9	56.5 ± 16.0	73.4 ± 10.7	62.1 ± 17.0	53.7 ± 19.6	10.245 (***p*** **= 0.036**)

^a^The percentages of voluntary admission at baseline are different from those in Table 1 because they are calculated for 92 patients at 8 years (6 patients died during follow-up).

Data are presented as mean ± SD or *n* (%). Total *n* varies for the different variables due to missing data. The percentage given for each variable refers to the total *n* available for that variable.

*p* values are results of Chi-square tests (categorical variables) for the comparison between adherence groups. Values in bold are significant at *p* < 0.05.

Note: GAF, Global Assessment of Functioning.

As seen in Table 4, a high proportion of patients whose adherence ‘improved from bad to good’ had either never used cannabis (50%, 11/23) or stopped using it during the study (38.9%, 9/23). Among the 40 patients with ‘always bad’ adherence, 20 (50%) continued to use cannabis, while 16 (38.32%) never used cannabis. Only 4 patients (11.8%) with ‘always bad’ adherence stopped using cannabis during the study, and there was a high frequency of alcohol use (75%) and other drug use (32.5%) in this subgroup.

In the multiple logistic regression, after controlling for type of first admission, patients who never used or stopped using cannabis during follow-up were more likely to have improved adherence than patients who continued using cannabis during the study (B = 2.174, OR = 8.794, *p* = 0.011). Similarly, after controlling for cannabis use, patients with involuntary first admission were more likely to have improved adherence than patients who had voluntary admission (B = 1.731, OR = 5.645, *p* = 0.047).

## Discussion

The two main findings of this study were that a longitudinal improvement in adherence was associated with involuntary first admission and with quitting cannabis use during follow-up in patients with recent psychotic episodes. Our results suggest that cannabis use and its withdrawal may be a mediator of adherence in FEP patients. However, the relationship could be bidirectional. Our findings imply that efforts should be made to help FEP patients quit cannabis use as this may improve their medication adherence and result in improved clinical and functional outcomes. This could be especially important in patients with involuntary admissions.

At first hospitalization, 74.4% of our study sample had bad adherence, which is consistent with previous reports of FEP patients [1]. It has been shown that FEP patients can provide a reasonable estimate of medication adherence [32].

In our study, 57.1% of the FEP patients had involuntary first admission to the psychiatric unit and all of these patients had bad adherence upon admission. This high level of involuntary admission is consistent with reports from other European countries that involuntary admissions for mental disorders have increased in recent years [33,34]. An important finding of our study is that treatment adherence improved from bad to good during 8 years of follow-up for 25% of the study sample, and that almost all (96%) of these patients had been hospitalized involuntarily at baseline.

Involuntary admissions were associated with improved adherence in the long term, and good adherence was associated with better functionality. Although they did not measure the relationship between involuntary admission and adherence, Priebe et al. [35] found that 40% of patients with involuntary first admission not only considered this therapeutic measure necessary but also these patients had better prognosis.

Adherence was related to functionality at the 8-year visit. The GAF mean scores show that patient functioning was generally poor at baseline and did not improve during follow-up. This is consistent with the Strauss–Carpenter scale scores at 8 years, which showed that 44.6% of patients were not working, 37.7% had been hospitalized during the previous year, and 15.2% had no social activity. Patients with bad adherence at 8 years had worse functionality than patients with good adherence at 8 years. In our sample of FEP patients, cannabis use was high at baseline (52% of patients), but fell to 29.3% at 8 years. Alcohol consumption rates also decreased from 95.9% to 56.5%. Our findings agree with other studies showing that FEP patients often have multiple substance use initially, and that about half of the patients stop or reduce cannabis use during treatment [36].

Consistent with a previous longitudinal study [18], we found a significant association between cannabis use and treatment adherence in FEP patients during long-term follow-up. The majority (20/27, 74%) of patients who continued to use cannabis over the 8-year follow-up period had consistently bad adherence, whereas only 17% (4/23) of the patients who stopped using cannabis were in the always bad adherence group. We have previously reported that FEP patients who stopped using cannabis during 8 years of follow-up had better functional outcomes and fewer negative symptoms than patients who continued using cannabis or who had never used cannabis [37]. Our present results extend this by showing a relationship between long-term cannabis use, poor functionality and the pattern of adherence. Likewise, Schimmelmann et al. [36] demonstrated that FEP adolescents who stopped using cannabis had better outcomes (CGI-S, GAF, remission of positive symptoms) than those who continued to use cannabis, although non-adherence did not explain the association between persistent cannabis use and worse outcomes. Persistent substance abuse is also a significant predictor of service disengagement [38], adding to the growing evidence that reduction of substance use should be a major focus of treatment in FEP patients.

The adherence pattern over 8 years of follow-up was associated with admission status at baseline (involuntary/voluntary) and the course of cannabis use over time, while alcohol use at first hospitalization was associated with baseline adherence, indicating that these variables may be early predictors of adherence. Consistent with this, Hill et al. [11] found that alcohol or substance misuse at inception predicted non-adherence at four years. However, Weiss et al. [14] reported that substance use at baseline did not predict future non-adherence, but the different findings may be because the patients were not inpatients or first-episode patients, and that substance use was mild and measured using a different scale.

Our study has several limitations. First, adherence was measured by self-report and is, therefore, subject to recall bias and possibly overestimation. Additionally, adherence was categorized as ‘bad’ or ‘good’, which is an over simplification of such a complex phenomenon. Second, the study sample was from the Vitoria region of Spain and the results may not be generalizable to FEP patients from other areas/regions. Another limitation is that we only included hospitalized patients; therefore, the findings are not applicable to FEP patients who do not require hospital admission, which can be up to 20% of patients [39]. Also, we examined the type of first hospital admission (voluntary/involuntary) as a factor associated with adherence, but did not consider the frequency of re-admissions during follow-up, which may be an important independent variable [40]. Moreover, we did not evaluate the patient‒doctor relationship, which could be a major factor influencing adherence [14]. Finally, our analyses were for patients with 8 years of follow-up data. Patients lost to follow-up may be non-adherent and have different outcomes and associations from those who remained in the study. Above all, as this study was not randomized, it is important not to assume causal relationships.

Recently, Martinez-Ortega et al. [40] stressed the need for prospective longitudinal investigations of the relationship between clinical variables, socio-demographic factors and voluntary/involuntary admission status of patients with mental disorders. The main strengths of our study are the prospective design and assessment of a consecutive series of FEP inpatients from a geographically-defined population over a long follow-up period of 8 years. Other strengths are that we repeatedly measured adherence and substance use at various time points during the study, giving us a picture of how they changed over the course of illness. We also used a wide range of standardized measures to gather information on other variables that may be involved.

## Conclusions

This study confirms there is a high rate of cannabis use among patients hospitalized with first-episode psychosis. More importantly, it shows an association between cannabis use and treatment adherence over an 8 year period, with better long-term adherence among patients who never used cannabis or who stopped using it. Adherence during follow-up is also better in patients with involuntary first admission. Since almost all of the patients with improved adherence over time had been admitted involuntarily at baseline, and many of these patients quit using cannabis, this shows the importance of involuntary admissions in FEP. Our findings imply that FEP patients with coexisting cannabis use are more likely to be non-adherent to medication and should be a target for interventions aimed at both stopping substance use and improving adherence. Such efforts include involuntary admissions and may result in improved clinical outcomes and patient functioning although further studies are needed.

## Competing interest

The authors have no competing interest that are directly related with this study.

## Authors’ contributions

BB and PV oversaw clinical management of the clinical dates of the patients and controls. SRA, IG and MS were the clinicians who participate in the recruitment, diagnosis and the management of the pharmacological treatment. CB and MMC participated in the design of the study and performed the statistical analysis. MH and BFC contributed in the review of the article. AGP and SB conceived of the study, and participated in its design and coordination and helped to draft the manuscript. All authors read and approved the final manuscript.

## Pre-publication history

The pre-publication history for this paper can be accessed here:

http://www.biomedcentral.com/1471-244X/13/326/prepub
